# Notes and News

**Published:** 1904-12-10

**Authors:** 


### Notes and News.

Princess Christian will preside at the annual meeting of the Mothers' Aid Society in Connection with Queen Charlotte's Hospital, on December 12th. This society fulfils a useful purpose of providing the more destitute patients with food, clothes, and coals, on returning to their homes.

The Royal Family are showing their sympathy with the movement to supply Windsor with a new hospital in a very practical manner. Towards the purchase of a new site the King has given £420 from the Windsor State Apartments Fund and £105 from his privy purse. The Queen gives £105 also, the Prince of Wales £50, and Prince and Princess Christian each give £25. Some very handsome contributions have already been received from the neighbourhood, and as Prince Christian is making a personal appeal, there is little doubt that the movement will be liberally supported.

The next Chemists' Exhibition will be held in Covent Garden Theatre from March 13th to 17th.

It is announced that a medical student at Oporto has died of the plague, contracted through pricking his finger with an instrument used in a post-mortem on a case of plague.

In the current number of *Truth* there is an expression of surprise that every member of the General Medical Council receives on an average £100 a year for attending meetings. After stating that the last session of the Council was consumed in "futile discussion and squabbling over more or less trumpery matters," our contemporary doubts if the Council justifies its very expensive existence from the point of view of the medical profession, out of whose pockets the necessary income is derived. The Editor of *Truth* seems to be unaware that the General Medical Council is charged with the protection of the public, and does not directly confer a single benefit on the medical profession. But, with its characteristic attitude towards the medical profession, the public leaves to them the honour of paying the bill.

By the death of the Earl of Hardwicke Queen Charlotte's Hospital loses a very valuable friend. He was chairman of the Committee of Management in the year 1898 and devoted considerable time to the institution, in which he was greatly interested.

Dr. F. Dittmar has been appointed medical inspector to the Local Government Board of Scotland in place of Dr. W. Leslie Mackenzie, who has been made medical member of the Board. Dr. Dittmar's present appointment is medical officer for Scarborough.

The report that comes from Gishiginsk, in Siberia, that the inhabitants of two villages have died of hunger, seems hardly credible in these times of rapid transit and civilisation. It is stated that two steamers with provisions were despatched to the stricken district, but failed to reach in time to save the starving population, whose distress is said to have been caused by a scarcity of fish.

The Army Council have decided that in future members of the Royal Army Medical Corps who have not qualified for warrant rank shall retire as soon as they are entitled to the minimum pension of that rank, or reach the age of 45, whichever happens first, and also those who have qualified at the age of 45, or on the completion of 21 years' service, whichever happens last, and provided that they have five years' service as warrant officer.

The Royal Waterloo Hospital for Children and Women will benefit to the extent of 2,000 guineas through the will of the late Mr. A. C. Crooke. This sum will be devoted to the endowment of two beds. The late Mr. James Smith, of Lee, has bequeathed £1,000 each to the Miller and the Seamen's Hospitals, Greenwich, and to the British Home for Incurables, £500 to the London Hospital, and smaller sums to numerous other charities.

Mrs. Close's scheme to establish farm homes in Canada for the upbringing of English pauper children is a bold and interesting one. The machinery would of necessity be of a complicated and colossal character; but if after careful consideration the experiment is possible of attempt, it should certainly be undertaken, if only to counteract some of the evils of overcrowding from which our urban population is markedly suffering without any immediate prospect of improvement.

Is the flower of charity to be choked by weeds? If not, those whose pleasure it has been to keep the garden in order must look to it well. A short while ago we warned our readers against an attempt which was then being made to advertise a newspaper under the guise of charity. A somewhat similar scheme has lately been started by the *Daily Express*, which is inviting monthly subscriptions by an offer to pay sixpence for each such subscription received towards a fund to "help London's starving thousands." However pure may be the motives of those who promote this type of "charity," they would better serve the interests of the poor if they were to allow the stream of their generosity to flow through direct channels.

